# Prediction and Analysis of Financial Default Loan Behavior Based on Machine Learning Model

**DOI:** 10.1155/2022/7907210

**Published:** 2022-09-20

**Authors:** Herui Chen

**Affiliations:** ^1^Chongqing Finance and Economics College, Chongqing 401320, China; ^2^School of Economics, Peking University, Beijing 100871, China

## Abstract

In recent years, the increase of customer loan risk and the aggravation of the epidemic have led to the increase of customer default risk. Therefore, identifying high-risk customers has become an important research hotspot for banks. The customer's credit is the standard to evaluate the loan amount and interest rate, and the ability to quickly identify customer information has become a research hotspot. Based on the bank credit application scenario, this paper realizes function extraction and data processing for customer basic attribute data and download transaction data. Then, a linear regression model with penalty and a neural network prediction model are proposed to improve the accuracy of bankruptcy assessment and achieve local optimization. In this way, the implicit risk prediction and control of customer credit are improved, and the default risk of bank loans is significantly reduced. According to the characteristics of the collected sample data, the most appropriate penalty linear regression prediction algorithm is selected and the experimental analysis is carried out to improve the risk management level of banks. The experimental results show that the improved logistic regression and neural network model has obvious advantages in the prediction effect for four models.

## 1. Introduction

As an important core of modern economy, the quality and management level of bank loans directly affect the economic system [1, 2]. Since loans are risky transactions of financial institutions, creditors or investors will undergo different levels of risk assessment before each loan transaction to assess whether creditors will repay the loans on time [3]. In the past, commercial banks usually used 5C classification to make subjective decisions when evaluating the credit risk of credit users [4]. They will assess and evaluate borrowers from five aspects: personal nature, credit constraints, solvency and market conditions, and credit services provided to customers. To a great extent, the judgment result depends on the subjective evaluation of risk appraisers in the evaluation process. From the perspective of internal risk control of banks, risk controllers may commit internal fraud. Due to the rapid change of market economy, this assessment method can no longer meet the needs of borrowers or the risk management requirements of commercial banks. We need to establish a scientific and effective explanation model to evaluate the reputation of credit customers, so as to minimize the default risk and maximize profits. If the global economy is to develop steadily and healthily, commercial banks must implement a scientific credit risk management system at the core of the economy [5].

By analyzing the transaction data of bank customers, the most basic credit information and many credit card data, we can obtain a lot of financial knowledge related to banking services. The prediction effect of financial risk in the abovementioned model is poor, especially the uncertainty in the prediction and judgment of customer credit and decision-making loan amount. The paper forecasts and judges the credit degree and decision-making loan quota of customers in banks. The proposed improved logistic regression and neural network method can achieve better prediction results in financial risk prediction. The second part of the paper describes the financial loan risk prediction algorithm, focusing on the non-balanced data, TF-IDF extraction method, and penalty regression method. The third part is the empirical analysis of commercial bank loan data. By comparing with other algorithms, the method proposed in this paper has a better effect.

## 2. Financial Loan Risk Prediction Algorithm

In the practical application of the linear regression model, the conventional least square method should be changed [6, 7]. The conventional least square method can only minimize the error of training data, and it is difficult to estimate all data. This section introduces an algorithm to overcome the least squares over-adaptation problem that is unfavorable to linear regression. Five indicators, such as CoVaR, cross-sectional VaR, absorption ratio, Granger causality index, and information spillover index, are used to measure systemic financial risks. The leading-lagging relationship among these five indexes and their forecasting ability to macroeconomy are investigated in detail. The results show that CoVaR, absorption ratio, Granger causality index, and information spillover index all have certain forecasting ability to macroeconomy in advance, but cross-sectional VaR has no forecasting ability; Granger causality index has a certain lead compared with other indicators, while absorption ratio and spillover index lag behind [8]. An unsupervised learning method is pretrained to automatically learn the distributed representation of data. Then, the model uses a supervised and fine-tuned deep neural network to classify and predict traders. The results of experimental evaluation confirm the effectiveness of the prediction model based on deep learning [9]. Aiming at the problem of unbalanced samples in the analysis of competitive intelligence, this paper proposes a method of identifying enterprise risk based on unbalanced samples, which takes the prediction of credit risk of financial enterprises as the breakthrough point. This method adopts intelligent analysis methods such as feature selection, unbalanced sample balance processing, and ensemble learning in the field of artificial intelligence analysis and provides solutions for enterprise risk identification in enterprise competitive intelligence under big data environment [10].

### 2.1. Unbalanced Data

Unbalanced records refer to records with great differences between different samples in records. Traditional classification algorithms treat all types of samples equally, and the generalization error is large [11].

#### 2.1.1. Resampling

Resampling mainly includes undersampling, oversampling, and weighted random sampling.

1. Undersampling. The method undersampling realizes class distribution by reducing most class samples [12]. This method easily loses the information of most class samples and reduces the classification effect of the model. However, previous studies have shown that the method under sampling has a good optimization effect on the model.
2. Oversampling. Contrary to the emphasis, oversampling focuses on several samples [13]. Oversampling is a way to balance the distribution of categories by adding multiple samples. In this paper, an improved SMOTE algorithm is proposed. The safety factor of several types of samples is analyzed, and then enhanced to ensure that the composite sample is closer to the sample with the highest safety factor, so that the noise and limit are blurred by the traditional SMOTE algorithm. Therapeutic SMOTE is a combination of therapy and smoothing therapy. The SMOTE algorithm firstly uses the CURE algorithm to cluster [14], adds a small number of samples to the original data, and then deletes the extractor. And it further generates noise and finally randomly generates new samples between the representative point and the center.
3. Weighted random samples [15]. Weighted random sampling is a random sampling method based on the weight of each sample in the original data set, in which the weight of each sample can be manually adjusted according to the experimental needs. By adjusting the larger weights of multiple samples, the sampling probability is higher. On the contrary, by adjusting the smaller weights of most samples, the sampling probability is lower, which can be improved.

#### 2.1.2. Cost-Sensitive Learning

A cost-sensitive method is introduced to increase the misclassification cost of some samples and reduce the misclassification cost of most samples [16]. Therefore, the model pays more attention to the performance under a small number of samples. Commonly used cost-sensitive functions include weighted transverse thrombus loss function and focus loss function.

*(1)Weighted Lateral Loss Function*. In the case of binary classification problems, the lateral loss function is defined as follows:

(1) $$\begin{array}{c} \begin{array}{c} Loss=-\frac{1}{N}\overset{N}{\underset{i=1}{\sum }}\left(y^{\left\{i\right\}}\;\log\;{\hat{y}}^{\left\{i\right\}}+\left(1-y^{\left\{i\right\}}\right)\log\;\left(1-y^{\left\{i\right\}}\right)\right) \end{array}, \end{array}$$

where *y* ^{*i*}^ represents the actual value of the *i*-th sample and ${\hat{y}}^{\left\{i\right\}}$ represents the predicted value of the *i*-th sample. The closer the predicted value is to the actual value, the smaller is the loss. The range of *y* is to deal with numbers between 0 and 1. It is used to represent the probability that the model is judged to be positive for a given input value.

The cross-sectional loss function can approximately measure the classification errors of two types of classification problems. The weighted cross-sectional loss function is defined as follows:

(2) $$\begin{array}{c} \begin{array}{c} Loss=-\frac{1}{N}\overset{N}{\underset{i=1}{\sum }}\left(\alpha y^{\left\{i\right\}}\;\log\;{\hat{y}}^{\left\{i\right\}}+\left(1-\alpha \right)\left(1-y^{\left\{i\right\}}\right)\log\;\left(1-y^{\left\{i\right\}}\right)\right) \end{array}, \end{array}$$

In the training process, we can constantly adjust *α*, adjust the cost of different classification samples, and make the model pay more attention to a few classification samples, so as to achieve the best classification effect.

(2) *Focus Loss Function*. The focus loss function is also an improvement of the cross entropy loss function, which is defined as follows:

(3) $$\begin{array}{c} \begin{array}{c} FL=-\frac{1}{N}\overset{N}{\underset{i=1}{\sum }}\left(\alpha y^{\left\{i\right\}}{\left(1-\hat{y}\right)}^{\gamma }lg{\hat{y}}^{\left\{i\right\}}+\left(1-\alpha \right)\left(1-y^{\left\{i\right\}}\right){\hat{y}}^{\gamma }lg\left(1-y^{\left\{i\right\}}\right)\right)\text{.} \end{array} \end{array}$$

In order to balance the factors, *α* pays more attention to a few samples, which brings high cost to the wrong classification of a few samples.

### 2.2. Extraction Using TF-IDF Method

TF-IDF algorithm is a very common extraction method [17, 18]. It is a statistical algorithm for weighted queries. In short, their task is to evaluate the importance of words to documents. In addition, TF-IDF method is very suitable for functional extraction of large amounts of data.

#### 2.2.1. Feature Extraction and Feature Engineering

Tests are needed to determine which attributes can be used for prediction. This process includes feature extraction and feature engineering. For example, in the spam filter question, enter the text of the e-mail message. Then, according to these attributes, spam and nonspam are distinguished.

Feature engineering is a process of sorting and combining functions to obtain more information [19]. Establish a loan authorization system, including feature extraction and feature engineering. Extracting features will determine which features of customers can be used to predict whether loans can be made [20]. Data such as previous loans and mobile app download history can be used to predict customer integrity.

After selecting some reasonable features, we need to train a prediction model and evaluate its performance. There must be pressure to improve the performance, but it is necessary to obtain and deploy the trained model quickly.

Modeling is also a process. At each startup, first select the function as the baseline. As a modern machine alarm algorithm, it usually trains 100–5000 different models and then chooses one model to use. If you do not want the model to be too simple, you do not want to give up performance, you do not want the model to be too complex, and you do not want the right problems to occur, you must choose the most appropriate model from the models with different complexities.

TF-IDF is a commonly used statistical method, which is usually used to evaluate the word meanings of documents in document sets or corpora. For example, search engines typically use different forms of TF-IDF weights to evaluate the relevance between a file and its user queries.

#### 2.2.2. Principle and Application of TF-IDF Algorithm

If the number of files with *T* elements in a Class *C* file is *m*, and the total number of files with *T* elements in other file types is *k*, then the number of files with *T* is obviously *n* = *m* + *k*. In this context, the term frequency (TF) refers to the frequency and number of times a particular word appears in a document. This number is normalized to a date number to prevent it from being transferred to a long file. For words in a specific file, the meaning is as follows:

(4) $$\begin{array}{c} \begin{array}{c} {tf}_{i,j}=\frac{n_{i,j}}{\sum_{k}n_{k,j}} \end{array}\text{.} \end{array}$$

By dividing the total number of documents by the number of documents that contain the word, you get the logarithm of the quota and the IDF for a specific word:

(5) $$\begin{array}{c} {i\;df}_{i}=\log\frac{\left|D\right|}{\left|\left\{j:t_{i}\in d_{i}\right\}\right|}\text{.} \end{array}$$

The accuracy rate is calculated as follows:

(6) $$\begin{array}{c} \begin{array}{c} Accuracy=\frac{TP+TN}{TP+TN+FN+FP} \end{array}\text{.} \end{array}$$

Download user application statistics, regard the application name downloaded by each user as a word, and change the weights of atypical and nonreference applications (such as WeChat applications) and representative applications (such as credit applications), so that they have a large number of downloads and a large number of downloads.

In the case of TF-IDF processing, when the user downloads app data, the words with lower frequency in the data are discarded first because these words have a small proportion and no attributes; next, we gave up more than 90% of the words. These words are frequent and atypical; after a series of edits, we finally selected the 3000 most critical application words as the words that can be used to predict credit default. In this record, we use cut acne to separate each application word and use the pound character as a separate word. In addition, we noticed that there are some duplicate users in the data that need to be re-edited to complete the extraction function.

### 2.3. Introduction of Penalty Regression Method

The linear regression method is a series of very useful prediction algorithms [21]. It comes from the commonly used least square method (OLS) proposed by Gauss and French mathematician Adrian Marie Legendre about 200 years ago. For example, a person's income can be predicted according to its size. According to its size, we can predict a person's income.

**Figure 1:**
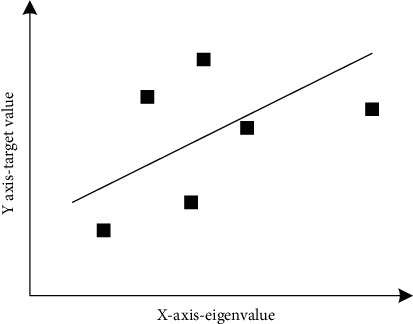
Ordinary least square fitting.

**Figure 2:**
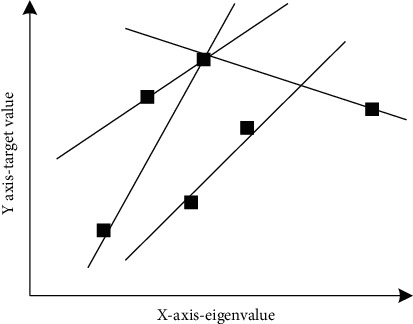
A straight line fitted with only two points.

Imagine a set of points, such as the points in Figure 1, but you cannot get all the points. The possible reason is that the cost of this case is too high to obtain all these data, just like the genomic data mentioned above. As long as there are enough people, you can isolate the genome of criminals, but the main problem is the cost. You did not get all the gene sequences. In fact, when you look at the effect of these combinations, you can choose two points in Figure 2 and imagine a straight line passing through them. Figure 2 shows the two possible pass-through points.

Take a straight line on a horizontal plane, and then push it up and down or rotate it to change its slope. It has nothing to do with the focus and inclination of the *x* axis. They can be changed individually. The two lines are connected into a straight line. The degree of freedom of a straight line can be expressed in a lumped equivalent way. All these methods of determining linear representation require two parameters.

Figure 2 shows six points fitted into a straight line (two degrees of freedom), in other words, six points correspond to two degrees of freedom. Looking for genes that can produce many human genes. The more genes you can choose from, the more data you need. In many cases, research projects with relatively reasonable budget can only be used in this case, and penalty regression linear regression is the best choice.

Penalty linear regression has the following characteristics: the training speed of the model is fast enough [21, 22], the important information of variables and the prediction speed in the operation process are fast enough, and the performance of various problems is reliable, especially for attribute matrix. These properties make the penalty linear regression method very effective.

### 2.4. Build a Loan Default Prediction Model Based on Big Data

This section will specifically describe the construction of linear penalty regression standard loan forecasting model based on big data.

#### 2.4.1. Loan Data Analysis and Processing

The data in this paper is divided into two parts. One part is the basic attributes of loan user data, including user number, user type, call number, mobile phone usage time, and monthly call charge. Although the client has described it in many ways, there are still some data fields missing. In the case of less data in the data set, the principle of basic completion of missing data should be adopted for manual completion to ensure the integrity of data.

Another part of the data is the data of the application downloaded by the user. Nowadays, people are willing to handle all kinds of things through mobile phones. There are many loan programs on the Internet, so the software downloaded by customers can also help analyze whether this person can get a bank loan.

#### 2.4.2. Construction of Loan Default Prediction Model Using Weighted Logistic Regression Algorithm

**Figure 3:**
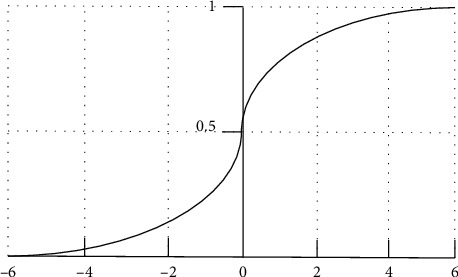
Principle of logistic regression algorithm.

Logistic regression is used to describe the results of linear regression (−∞, ∞) on (0, 1) by S-shaped function [23]. This will cause *Y* to take a value other than 0 or 1. Logistic regression uses a function to normalize the *Y* value so that the *Y* value is in the interval (0, 1). This function is called a logical function, also known as an S-shaped function. The principle of logistic regression algorithm is shown in Figure 3, and the function formula is as follows:

(7) $$\begin{array}{c} \begin{array}{c} g\left(z\right)=\frac{1}{1+e^{-z}} \end{array}\text{.} \end{array}$$

Logistic regression is a function of *e* in the form *y* = 1/(1 + *e*(−*x*)); that is,

1. When *x* > 0, with the increase of *x*, *y* approaches 1 quickly
2. If *x* < 0, *y* approaches rapidly as *x* decreases
3. If *x* *=* 0, *y* = 1/2

Because of this characteristic of logistic regression (continuous between 0 and 1), it is used to evaluate whether the learning algorithm is correct.

In addition to the right and wrong results, the advantage of logistic regression is that it can also help to find out how far away from the right results, thus guiding the results in the right direction. Therefore, it is usually used in conjunction with the Steig algorithm.

Combined with the above characteristics, we can see that the characteristics of logistic regression algorithm are very suitable for predicting the credit default of bank customers [24]. However, combined with practice, it can be concluded that loan default is very isolated and the default rate is relatively low. Raw unbalanced data can be programmed into more balanced data for model training and prediction.

#### 2.4.3. Introducing Neural Network Model

**Figure 4:**
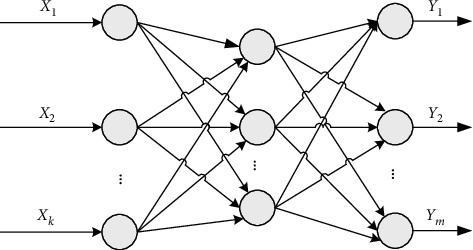
Neural network model.

At present, most artificial neural network models adopt feed-forward neural network with error reverse transmission, that is, the BP neural network. Its structure is relatively simple, its parallelism is strong, and it has strong nonlinear mapping ability, so it is widely used in classification and prediction problems. When solving the classification problem of *K* class, for any sample (*x*_*i*_, *y*_*i*_), make *y*_*i*_: = *y*_*i*_, where *y*_*i*_={*y*_*i*_^1^, *y*_*i*_^2^,…, *y*_*i*_^*k*^}, $y_{i}=\left\{\begin{array}{c} 1,y_{i}=y_{i}^{k}\\ 0,y_{i}\neq y_{i}^{k} \end{array}\right.$ . A new sample (*x*_*i*_, *y*_*i*_) is obtained for training and testing the model. The neural network model is shown in Figure 4.

The BP neural network has three-layer structure: input layer, hidden layer, and output layer. For the *i*-th sample (*x*_*i*_, *y*_*i*_), let the input layer be *a*_1_ = [*a*_1_, (*x*_*i*_)^Τ^] and the number of neurons in the hidden layer be L. In this experiment, by comparison, it is found that the number of neurons in the hidden layer is selected by formula (8):

(8) $$\begin{array}{c} L=\sqrt{m+n}+\alpha \text{.} \end{array}$$

The inputs of the hidden layer are *z*_2_=*a*_1_ *θ*_1_, where *θ*_1_ ∈ *R* ^(*n* +1) ×*L*^ and the outputs are *a*_2_=[*a*_2_^0^, *g*(*z*_2_)]. The input of the output layer is z_3_=*a*_2_ *θ*_3_, where *θ*_2_ ∈ *R* ^(*L* +1) ×*K*^ and the output is *a*_3_=*g*(*z*_3_), then the prediction function *h*_*θ*_(*x*_*i*_)=*a*_3_.

(9) $$\begin{array}{c} J\left(\theta \right)=\frac{1}{m}\overset{m}{\underset{i=1}{\sum }}\overset{K}{\underset{k=1}{\sum }}\left[-y_{i}^{k}\log{\left(h_{\theta }\left(x_{i}\right)\right)}_{k}-\left(1-y_{i}^{k}\right)\log{\left(1-h_{\theta }\left(x_{i}\right)\right)}_{k}\right]+\frac{\lambda }{2m}\left[\overset{n}{\underset{i=1}{\sum }}\overset{L}{\underset{l=1}{\sum }}{\left(\theta_{i,l}^{1}\right)}^{2}\right.+\overset{L}{\underset{l=1}{\sum }}\overset{K}{\underset{k=1}{\sum }}{\left(\theta_{i,l}^{2}\right)}^{2}\text{.} \end{array}$$

For the *i*-th sample (*x*_*i*_, *y*_*i*_), the output layer error *δ*_3_ = *y*_*i*_ − *a*_3_. When the offset of *θ*_2_ in *θ*_0_^2^ is removed, *δ*_2_ = *δ*_3_(*θ*_2_)^Τ^ *∗g*′(*z*_2_) can be obtained.

The construct cost function is as follows:

(10) $$\begin{array}{c} \left\{\begin{array}{c} \Delta_{1}≔=\Delta_{1}+{\left(a_{1}\right)}^{T}\delta_{2}\text{,}\\ \Delta_{2}\Delta =\Delta_{2}+{\left(a_{2}\right)}^{T}\delta_{3}\text{.} \end{array}\right. \end{array}$$

The cumulative error formula is as follows:

(11) $$\begin{array}{c} \frac{\partial J\left(\theta \right)}{\partial \theta_{ij}^{1}}=\left\{\begin{array}{cc} \frac{1}{m}{∆}_{ij}^{1}\text{,} & \left(j=0\right)\text{,}\\ \frac{1}{m}{∆}_{ij}^{1}+\frac{\lambda }{m}{∆}_{ij}^{1}, & \left(j\neq 0\right)\text{,} \end{array}\right.\\ \frac{\partial J\left(\theta \right)}{\partial \theta_{ij}^{2}}=\left\{\begin{array}{cc} \frac{1}{m}{∆}_{ij}^{2}\text{,} & \left(j=0\right)\text{,}\\ \frac{1}{m}{∆}_{ij}^{2}+\frac{\lambda }{m}{∆}_{ij}^{2}\text{,} & \left(j\neq 0\right)\text{.} \end{array}\right. \end{array}$$

Finally, the optimization target $\min_{\theta }\;J\left(\theta \right)$ is obtained. After the model parameter *θ* is obtained by optimization algorithm, the predicted output $\hat{y}=h_{\theta }\left(x\right)$ for the input *x* of any input sample.

#### 2.4.4. Introducing Penalty Coefficient to Obtain Optimal Solution

Under the influence of this algorithm, the machine cannot independently identify the local or global functions in the learning process. Therefore, after training, the machine can not only receive global features of data but also receive some local functions. This is called universality and chemistry. These two problems are getting more and more serious, which is the biggest problem of renewable energy. The so-called equipment means that the training is too thorough, and all the characteristics of the data contained in the sample are checked.

The basic principle of this method is to limit machine learning and not make the features of machine learning deep enough, so as to reduce the possibility of local features and error features of machine learning, thus improving the accuracy of detection. Therefore, we introduce an adjustment (penalty coefficient) to adjust logistic regression. Regulation, L1 and L2 are regulatory concepts, also known as sanctions, which are elements added to the loss function to limit model parameters and prevent model over-adaptation. In this article, we use L1 control time and L2 control time to compare experiments to find the best solution. First, L1 regulation and L2 regulation are penalties as a function of loss. For linear regression models, formula (12) is the loss function of Lasso regression in Python. Formula (13) is the loss function of the Ridge regression in Python:

(12) $$\begin{array}{c} \begin{array}{c} \min_{w}\frac{1}{2n_{samples}}{\left\|X_{w-y}\right\|}_{2}^{2}+\alpha {\left\|w\right\|}_{1} \end{array}\text{,} \end{array}$$

(13) $$\begin{array}{c} \begin{array}{c} \min_{w}{\left\|X_{w-y}\right\|}_{2}^{2}+\alpha {\left\|w\right\|}_{2}^{2} \end{array}\text{.} \end{array}$$

In general regression analysis, regression *W* represents the characteristic coefficient. From the above formula, we can see that the concept of supervision involves coefficients (constraints). The preadjustment factor is added, usually as α. Of course, it is sometimes used to represent coefficients that will be set by the user.

Generally speaking, L1 rule can create scarcity matrix, which means that we can use frugality model for feature selection. Second language rules can prevent over-adaptation of models. In some cases, L1 can also prevent over-assembly.

(1) L1 *Regularization and Feature Selection*. Suppose we have the following loss function with L1 regularization, such as formula:

(14) $$\begin{array}{c} \begin{array}{c} J=J_{0}+\alpha \sum_{W}\left|W\right| \end{array}\text{.} \end{array}$$

It is an important task for machine learning to find the minimum value of loss function by some methods.

**Figure 5:**
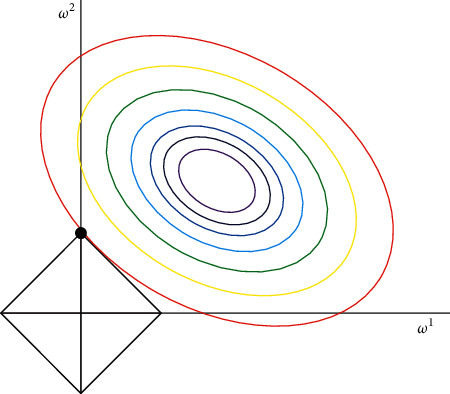
F1 regularization.

As shown in Figure 5, the coefficient *α* regularization can well control the size of the graph. The smaller the *α* curve *l*, the larger the curve *l* (the black box in the above Figure 5), the larger the *α* graph, the smaller the graph *l*, so the black box is only a point after the origin. This is the most appropriate value (W1, W2) = (0, *W*). *W* can take a very small value.

**Figure 6:**
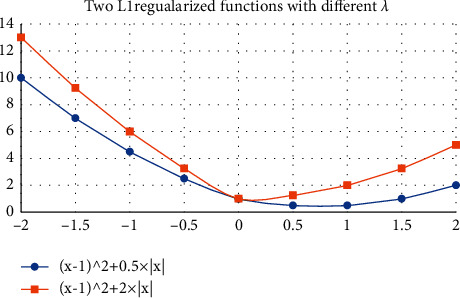
Selection of regularization parameters.

For L1 regularization parameter, under normal circumstances, the larger the input, when the parameter is 0, the penalty function can get the minimum value. Let us take a simple example and assume the following cost function with L1 regularization term, such as formula (15):

(15) $$\begin{array}{c} \begin{array}{c} F\left(x\right)=f\left(x\right)+\lambda {\left\|x\right\|}_{1} \end{array}\text{,} \end{array}$$

where *X* is the parameter to be estimated, corresponding to *W* and 0 above. It should be noted that L1 regularization cannot be derived in some places, and if the input is large enough, if *x* = 0 is to the minimum value, then *f* (*x*) can be taken, as shown in Figure 6:

When *x* = 0, the larger the value, the easier it is to get the minimum value of *f* (*x*).

(2) L2 *Regularization and Over-Fitting*. Suppose there is a loss function with L2 rule, such as the formula:

(16) $$\begin{array}{c} \begin{array}{c} J=J_{0}+\alpha \sum_{w}w^{2} \end{array}\text{.} \end{array}$$

**Figure 7:**
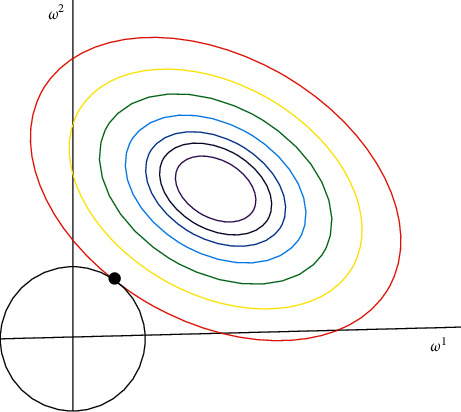
L2 regularization.

We can also draw a graphical representation of them on a two-dimensional plane, as shown in Figure 7.

In the linear regression equation, if the parameters are very large, even if the data moves a little, it will have a great influence on the results. However, if the parameter is small enough, slightly moving the data will not affect the result. Professionally speaking, the robustness is very strong, and L2 adjustment can receive small parameters.

Assuming that the required parameter is 0, *h*_*θ*_(*x*) is our hypothetical function, and the cost function of linear regression is as follows:

(17) $$\begin{array}{c} \begin{array}{c} J\left(\theta \right)=\frac{1}{2m}\overset{m}{\underset{i=1}{\sum }}{\left(h_{\theta }\left(x^{\left(i\right)}\right)-y^{\left(i\right)}\right)}^{2} \end{array}\text{.} \end{array}$$

Therefore, in the gradient descent method, the iterative formula used to calculate the parameter *θ* in the final iteration is as follows:

(18) $$\begin{array}{c} \begin{array}{c} \theta_{j}≔\theta_{j}-\alpha \frac{1}{m}\overset{m}{\underset{i=1}{\sum }}\left(h_{\theta }\left(x^{\left(i\right)}\right)-y^{\left(i\right)}\right)x_{j}^{\left(i\right)} \end{array}\text{.} \end{array}$$

If you add the regular element L2 after the initial cost function, the iteration formula becomes the following:

(19) $$\begin{array}{c} \begin{array}{c} \theta_{j}≔\theta_{j}\left(1-\alpha \frac{\lambda }{m}\right)-\alpha \frac{1}{m}\overset{m}{\underset{i=1}{\sum }}\left(h_{\theta }\left(x^{\left(i\right)}\right)-y^{\left(i\right)}\right)x_{j}^{\left(i\right)} \end{array}\text{.} \end{array}$$

The regularization parameters are included in the formula. Multiply *θ*_*j*_ by a factor less than 1, so *θ*_*j*_ can continue to decrease, so *θ*_*j*_ usually decreases.

For the L2 regularization parameter, the larger the input, the faster the attenuation of *θ*_*j*_. For another understanding, please refer to Figure 7. The larger the input of the formula, the smaller the radius of the L2 circle. If the final cost function is the maximum, its parameters will also become very small.

In order to solve the problem of credit default prediction caused by extremely unbalanced data in this paper, the training and prediction results of the model are easy to adjust. Therefore, L2 regulation period is more suitable for analysis. In the experiment, the first language and the second language adjust the concept of training and learning through the experiment to choose the most appropriate penalty coefficient.

## 3. Empirical Analysis of Loan Data of a Commercial Bank

### 3.1. Experimental Data

The original dataset shown in Table 1 has a relatively large dimension and sample size, and the original dataset does not contain any category identifiers. The table also shows that most attribute values have no data, and the maximum skip rate is even as high as 35%. Therefore, we need to use some appropriate data filler to populate the data for each missing attribute value.

**Figure 8:**
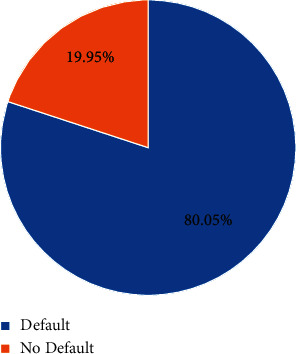
Sample category distribution.

There are 656,000 pieces of data in the dataset, and the number of nondefault samples is 640,390, accounting for 80.05%; there are 159,610 default samples, accounting for 19.95%, and the ratio of the two types of samples is 4 : 1. The distribution of samples is shown in Figure 8.

### 3.2. Experimental Data Preprocessing

In the complete raw dataset that we can get, it is obvious that there is no category label attribute. Therefore, if we make a systematic analysis from the perspective of data, we can clearly see that the bank credit data is only a cluster dataset. Credit quality can be classified according to the risk level of loans, and credit quality can be classified according to its risk level, which can be divided into four types: normal, concerned, secondary doubt, and loss. This grade is collectively called nonperforming loans, and we can also find these attributes suspiciously from the original bank credit data. At this point, we can generate the tag class attribute from the credit year attribute and combine these five attributes together, calling them “default.”

However, due to the long age distribution of the bank loan data set, it can be traced back to 2004 to 1979. If we use it suddenly, it will lead to two problems: (1) under the new Basel Accord and the guiding principle of credit classification proposed by the State Bank of China, the credit data around 1998 may have more obvious changes, which will have a significant impact on the final forecast results; (2) the adaptability of the model cannot solve such a long time period, thus reducing the robustness of the prediction model.

In view of these two problems, after the previous analysis, we use the credit data of the last three years as a sample set to predict. According to the credit data in the last three years, we can directly apply the New Basel Accord and the standard forecasting rules of the People's Bank of China to determine whether customers have credit default.

In this article, we can eliminate the following three types of attributes through domain-related knowledge:

1. Customer name and description marked on each credit: user name, industry, user credit, user ID, credit year, inquiry 1, start date, and inquiry 2 (the excluded part is only the basic description of the borrower and does not affect the final result)
2. According to the definition of default before 1998: normal balance, expected balance, and bad debts
3. There is only one value for this attribute: for fixed assets acquired through finance leases, deposits are used as collateral. After our data excludes, analyzes and ranks all attributes unrelated to domain knowledge, we use credit data sets. Many data losses have been confirmed.

In order to ensure the accuracy of the forecast, we first process the received bank user data. For training sets and validation sets, the missing data is the zero value of customer information. As an alternative, we use −99999 or 0. We collected 36 basic characteristics related to the basic attributes of customers. Some functions are missing on a large scale. Therefore, we decided to reject these useless attributes before forecasting, so as not to interfere with the forecasting results. Finally, we compare these basic data with them. Combined with the above customer application data, a complete dataset is created, which can be used to predict credit default.

### 3.3. Experiment and Analysis

#### 3.3.1. Experimental Indicators

There are many indexes to evaluate the accuracy of logistic regression prediction algorithm. In this paper, a variety of indicators are used to evaluate, namely, F1, confusion matrix, accuracy, recall, and accuracy.

#### 3.3.2. Comparative Experiment and Result Analysis

*(1)Comparative Test Devices*. In order to test the selection of different penalty coefficients and the difference of algorithm weights, this experiment forms four groups of comparative experiments. There are not 10000 data in the whole data set, 8000 data are used as training sets of prediction models, and 2000 data are used as test sets of prediction models. We compare and analyze the results using the penalty coefficients L1, L2, and an innumerable number of weighted permutations:

1. The penalty coefficient L1 is accepted, and the algorithm is not weighted
2. The algorithm should be weighted by the penalty coefficient L1
3. The penalty coefficient L2 is accepted, and the algorithm is not weighted
4. Accept the penalty coefficient L2 and weigh the algorithm

(2) *Experimental Results*. The above four situations were checked, and the test results of different comparative tests are shown in Table 2.

**Figure 9:**
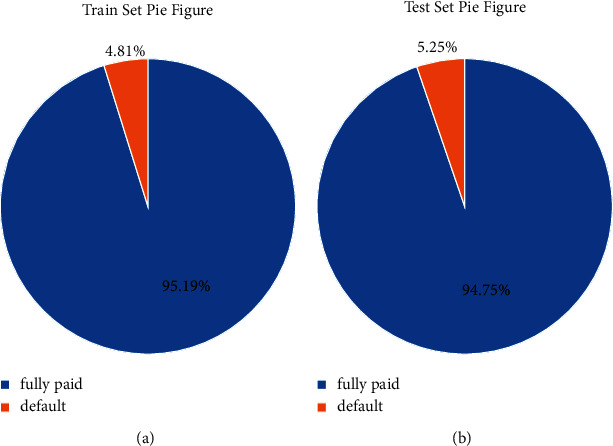
Penalty coefficient L1 and weighted results predict the correct proportion: (a) proportion of correctly predicted training sets; (b) proportion of correctly predicted test sets.

The test results in Table 2 show that no matter what the penalty coefficient L1 or L2 is, as long as the accuracy, recall rate, and F1 are all 0, the credit data cannot be well predicted. Compared with the weighted results, the accuracy and F1 obtained using the L2 penalty coefficient are significantly higher than those obtained using the L1 penalty coefficient. The result obtained by using the L2 penalty coefficient is better than that obtained by using the L1 penalty coefficient. The pie chart of training results and test results is shown in Figure 9.

Figure 9 shows that the prediction success rate of the test set is 94.75%, which is almost equivalent to the original accuracy rate of the training set of 95.19%. This is a relatively successful prediction model.

### 3.4. Comparative Test and Analysis

In addition to the above experiments, this paper also compares three traditional experimental models, logistic regression, decision tree, and AdaBoost, with the model proposed in this paper.

After data preprocessing, there are 79820 experimental data in this paper, and each data includes 39 features. In the experiment, 80% is used as the training model of the training group, and the remaining 20% is used as the model performance extraction of the test group. In order to verify the effectiveness of the AdaBoost model based on price sensitivity, this paper constructs four different models of logistic regression, decision tree, conventional AdaBoost, and the proposed prediction model in the preprocessing data, and compares the prediction effect and generalization performance of the four models horizontally.

**Figure 10:**
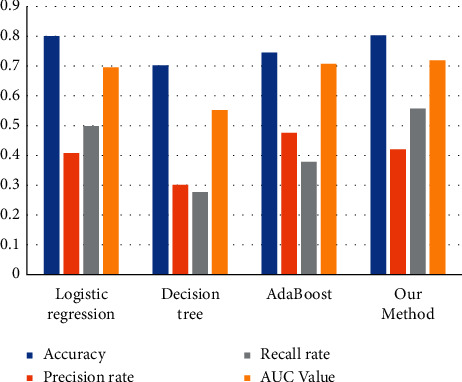
Performance comparison of different models.

The logistic regression model is easy to explain, and the algorithm principle is easy to understand, but as a representative of the linear model, logistic model is prone to multicollinearity. Logistic model has a wide range of application scenarios, and it is also common in the field of financial wind control. Because logistic regression is a generalized linear model, the model is easily affected by the characteristic dimension, which makes the model biased towards the characteristics with large absolute value, so this paper normalizes the data before building the logistic model. By constructing a logistic regression model, the prediction results of the proposed model and the most common wind control model can be compared. The decision tree model is the basic learner of the prediction model proposed in this paper, and the effect of model integration can be observed by building the decision tree model. By constructing the traditional AdaBoost model, the effect of the improved model can be obtained intuitively. Some evaluations of the test set of the four models are shown in Figure 10.

It can be seen from Figure 10 that the prediction effect of the decision tree model is the worst among the four models. The accuracy of the logistic regression model and the prediction model given in this paper reaches 80% and 80.27%, respectively, which is obviously better than that of the decision tree model and the conventional AdaBoost model. Compared with the traditional AdaBoost model, the prediction model proposed in this paper is reduced due to the addition of the price-sensitive penalty factor, but the improved AdaBoost model is more effective in dealing with the problem of data imbalance, and the AUC value reaches 71.87%, which is better than the other three models.

## 4. Conclusion

With the rapid development of socialist market economy in China, the demand of individual customers for credit transactions has also increased significantly, but there are many hidden dangers behind this phenomenon. For example, if a customer wishes to obtain bank credit for his business, the bank must also provide credit transactions to the customer to make a profit. However, in order to prevent banks from issuing successful loans to control inefficient borrowing rates, it is particularly important for banks to assess whether customers do not use huge and sensitive customer data. Therefore, this paper mainly provides solutions to the common credit default problems in finance and banking industry, using existing data to predict customer credit default, and proposes an improved penalty linear regression prediction algorithm based on weight. The main research results of this paper are as follows.

In this paper, a weight-based prediction algorithm model is proposed. By weighting the extremely unbalanced customer stem data and then extracting the app data downloaded by customers with TF-IDF function, it can be used for prediction. Combined with the previous basic data, it can be used as a useful dataset for default prediction of customer credit. Secondly, the logistic regression algorithm is used to build a customer credit default prediction model, and the penalty coefficient is introduced into the algorithm to prevent over-adaptation through model training. This model not only solves the problem that the default data of credit itself is difficult to predict and unbalanced but also solves the problem that the serious over-adjustment affects the prediction results in the process of model design. Comparative experiments show that the L2 penalty coefficient is the best in the credit default prediction model, which can improve the accuracy of the prediction results, thus obtaining the best prediction results, which has important reference value for predicting whether customers will avoid credit default.

## Figures and Tables

**Table 1:** Data set attribute description.

Dataset	Size	Attribute number	Category
Bank loan data	45596	38	None

**Table 2:** Experimental results under four conditions.

Test	Matrix	Precision rate	Recall rate	Accuracy	*F*_1_
L1 unweighted	[[1893 2]	0.0000	0.0000	0.9465	0.0000
	[105 0]]				

L2 unweighted	[[1894 1]	0.0000	0.0000	0.9470	0.0000
	[105 0]]				

L1 weighted	[[1194 701]	0.0896	0.6571	0.6315	0.1577
	[36 69]]				

L2 weighted	[[1252 643]	0.0918	0.9190	0.6585	0.1599
	[40 65]]				

## Data Availability

The raw data supporting the conclusions of this article will be made available by the authors, without undue reservation.
